# Biosynthetic and Synthetic Strategies for Assembling Capuramycin-Type Antituberculosis Antibiotics

**DOI:** 10.3390/molecules24030433

**Published:** 2019-01-25

**Authors:** Ashley L. Biecker, Xiaodong Liu, Jon S. Thorson, Zhaoyong Yang, Steven G. Van Lanen

**Affiliations:** 1 Department of Pharmaceutical Sciences, College of Pharmacy, University of Kentucky, Lexington, KY 40536, USA; ashley.arlinghaus@uky.edu (A.L.B.); xiaodong.liu@uky.edu (X.L.); jsthorson@uky.edu (J.S.T.); 2 Institute of Medicinal Biotechnology, Chinese Academy of Medical Sciences and Peking Union Medical College, Beijing 1000050, China; zhaoyongy@163.com

**Keywords:** natural product, nucleoside, antibiotic, MraY, bacterial translocase I, *Mycobacterium tuberculosis*

## Abstract

*Mycobacterium tuberculosis (Mtb)* has recently surpassed HIV/AIDS as the leading cause of death by a single infectious agent. The standard therapeutic regimen against tuberculosis (TB) remains a long, expensive process involving a multidrug regimen, and the prominence of multidrug-resistant (MDR), extensively drug-resistant (XDR), and totally drug-resistant (TDR) strains continues to impede treatment success. An underexplored class of natural products—the capuramycin-type nucleoside antibiotics—have been shown to have potent anti-TB activity by inhibiting bacterial translocase I, a ubiquitous and essential enzyme that functions in peptidoglycan biosynthesis. The present review discusses current literature concerning the biosynthesis and chemical synthesis of capuramycin and analogs, seeking to highlight the potential of the capuramycin scaffold as a favorable anti-TB therapeutic that warrants further development.

## 1. Introduction

Tuberculosis (TB), primarily caused by the bacterial pathogen *Mycobacterium tuberculosis (Mtb)*, is one of the oldest and deadliest infectious diseases known to humans. The World Health Organization (WHO) has recently reported that TB affected approximately 10 million people and killed roughly 1.3 million people in 2017 alone [1,2,3,4]. Although global TB-related mortality rates have steadily declined since the turn of the millennium, multidrug-resistant (MDR), extensively drug-resistant (XDR), and totally drug-resistant (TDR) strains pose significant threats to this progress [1,3,5,6,7,8,9]. Consequently, there is an urgent need for discovering new therapeutics to treat TB, which will in part help to offset the diminishing utility of the antibiotics currently used to treat TB. Capuramycin-type nucleoside antibiotics, which represent one such possible new class, are natural products that were re-discovered in the early 2000s and have significant promise for drug development as anti-TB therapeutics. This review summarizes the discovery of the capuramycin-type nucleoside antibiotics; progress toward defining their biosynthetic pathway; and synthetic, semisynthetic, and biocatalytic studies aimed at generating analogs with potentially improved therapeutic value. For an in depth description of *Mtb* and tuberculosis, including the major challenges in drug discovery and development, we refer the reader to several excellent reviews [10,11,12,13,14] and publications cited herein.

## 2. Discovery, Mechanism of Action, and Structure of Capuramycin-Type Nucleoside Antibiotics

**Figure 1:**
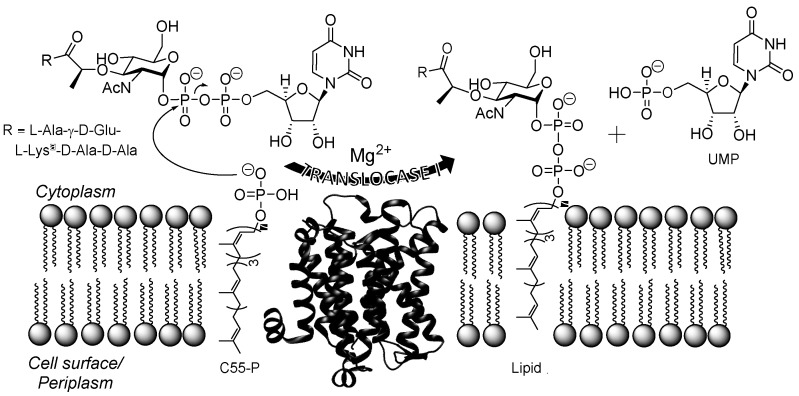
Depiction of the translocase I (TL1)-catalyzed reaction. TL1 transfers phospho-MurNAc-pentapeptide to C55-P to produce UMP and Lipid I. The Mg^2+^-dependent translocase I-catalyzed reaction occurs in the cytoplasm of the cell. The structure of TL1 from *Aquifex aeolicus* (Protein Data Bank ID: 4J72; [18]). ^a^l-Lys or l-diaminopimaleic acid (l-Dap) is found at this position depending on the genus of bacteria.

Bacterial translocase I (annotated as MraY in *Escherichia coli*, MurX in *Mtb*, and herein simply abbreviated as TL1) is an unusual transmembrane enzyme that catalyzes the first committed membrane step of peptidoglycan biosynthesis [15,16,17,18,19]. TL1 transfers phospho-*N*-acetylmuramoyl-pentapeptide (phospho-MurNAc-pentapeptide) onto the lipid carrier, undecaprenyl phosphate (C55-P), to produce uridine-5′-phosphate (UMP) and lipid I (Figure 1) [20]. This catalytic activity has been shown to be essential for the survival of Gram-positive, Gram-negative, and mycobacteria including *Mtb* [21,22,23]. In the early 2000s, Sankyo Co. (now Daiichi-Sankyo) recognized the potential of TL1 as an antibiotic target and developed a high-throughput screen aimed at identifying compounds, particularly those from bacterial extracts, that specifically inhibit TL1 activity in vitro [24,25,26,27,28,29,30]. A series of related nucleoside natural products were isolated from this screen and confirmed to potently inhibit TL1 with 10 to 38 nM IC_50_ values [25,28,30]. Additional studies revealed a select set of the nucleoside-containing natural products also have antibacterial activity as expected, including activity against Gram-positive bacteria, such as *Streptococcus pneumonia* [25,28], and most species of mycobacteria, including *Mtb* [31]. Importantly, a representative TL1 inhibitor with anti-TB activity in vitro was shown to be effective in a murine model of TB infection with no detectable toxicity [31]. 

**Figure 2:**
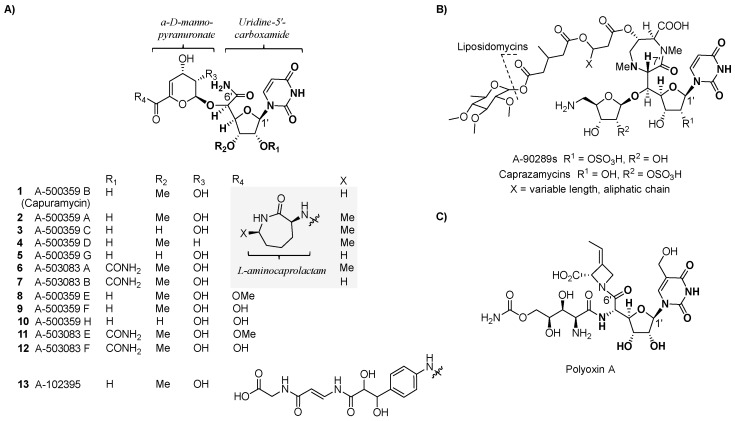
Structures of high-carbon sugar nucleosides. (**A**) Structure of the three major groups (A-500359s, A-503083s, and A-102395) of capuramycin-type nucleoside antibiotics isolated from different bacterial strains. (**B**) Structure of representative 5′-*C*-glycyluridine (GlyU)-containing nucleoside antibiotics. (**C**) Structure of the antifungal compound polyoxin A.

Three related groups of nucleoside-containing natural products were discovered by Sankyo Co. and named A-500359s (Figure 2A, **1** to **5, 8** to **10**), produced by *Streptomyces griseus* SANK 60196; A-503083s (**6**, **7**, **11**, and **12**), produced by *Streptomyces* sp. SANK 62799; and A-102395 (**13**), produced by *Amycolatopsis* sp. SANK 60206. Structural elucidation of A-500359s revealed three distinct chemical components: A uridine-5′-carboxamide (CarU), an unsaturated α-d-*manno*-pyranuronate, and l-aminocaprolactam (l-ACL). A-500359 B (**1**) was shown to be identical to a known natural product named capuramycin, which was discovered in 1986 from *Streptomyces griseus* 446-S3 as part of a search for antibacterial agents [32,33]. Hence, we have termed the three groups as capuramycin-type antibiotics. The A-503083s differ from the A-500359s by the presence of a 2′-*O*-carbamoyl group, which has a minimal effect on TL1 inhibition and anti-TB activity. For both A-500359s and A503083s, deaminocaprolactam derivatives **8** to **12** were isolated from the producing strains, and these analogs were shown to have significantly reduced inhibitory activity against TL1 as well as reduced antibiotic activity. Nucleoside **13** differs from the other two groups by containing an arylamine-linked polyamide chain instead of the l-ACL. This substitution imparts **13** with the most potent TL1 inhibition (IC_50_ = 10 nM) of the three groups but effectively eliminates all antibiotic activity including against *Mtb*.

The shared CarU and 4,5-dehydro-d-*manno*-pyranuronate of the capuramycin-type nucleoside antibiotics are structural components not found in other natural products [34,35]. The former is categorized as a high-carbon sugar nucleoside since the furanoside contains six contiguous carbons in place of the d-ribose of the canonical uridine nucleoside. Several other, structurally distinct high-carbon sugar nucleosides are known, including another category of TL1 inhibitors highlighted by a high-carbon sugar nucleoside of seven contiguous carbons, 5′-*C*-glycyluridine (GlyU). The GlyU-containing TL1 inhibitors, represented by the liposidomycin-type nucleoside antibiotics that includes A-90289s and caprazamycins (Figure 2B), also have anti-TB activity in addition to efficacy against other bacteria [36,37]. Another example of a group of natural products containing a high-carbon sugar nucleoside is the polyoxins (Figure 2C), which contain six contiguous carbons in place of the d-ribose of the canonical uridine [38]. In contrast to CarU, however, C-6′ of the polyoxin nucleoside is a carboxylate that is part of a terminal α-amino acid. Unlike CarU- and GlyU-containing nucleoside antibiotics, polyoxins do not inhibit TL1 but instead have antifungal activity due to selective inhibition of chitin synthase [39]. 

## 3. Biosynthesis

### 3.1. Investigation of Precursors 

**Figure 3:**
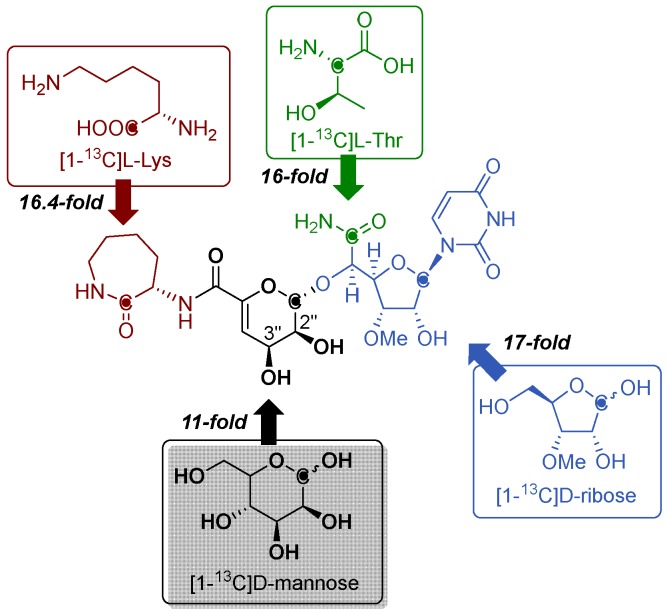
Metabolic origin of A-500359s and A-503083s as determined through feeding experiments using the indicated isotopically labeled precursors.

The biosynthesis of capuramycin-type antibiotics was first interrogated using feeding experiments with isotopically labeled precursors in 2003 with the goal of elucidating the metabolic origin of each structural component [27]. A convergent biosynthetic approach was predicted from CarU, α-d-*manno*-pyranuronate, and l-ACL components, which were likewise proposed to be derived from uridine, d-mannose, and l-Lys, respectively. Using A-500359s as the model capuramycins, a 17-fold enrichment at the C-1′ position using [1-^13^C]d-ribose, 11-fold enrichment of the C-1′′ position using [1-^13^C]d-mannose, and a 16-fold enrichment of the C-1′′′ position using [1-^13^C]l-Lys were found (Figure 3). The precursor of the C-5′ carboxamide of CarU, a unique feature of this family of nucleoside antibiotics as previously noted, was not readily apparent based on biochemical precedence. However, it was initially observed that A-500359s and polyoxins have high-carbon sugar nucleosides with a C-6′ carbonyl functional group [27,38]. Since the origin of the C-5′ carboxylate of polyoxin was previously assigned to phosphoenolpyruvate on the basis of isotopic enrichment studies using [3-^13^C]pyruvate [40], the C-5′ carboxamide of A-500359s was proposed to be derived from phosphoenolpyruvate in a similar manner. Feeding with sodium [3-^13^C]pyruvate, however, only yielded a three-fold enrichment of the C-6′ position of **1** [27]. This relatively low enrichment was therefore consistent with a different biosynthetic precursor for the C-5′ carboxamide in CarU, which was ultimately determined to be l-α-Thr (vide infra). Finally, an eight-fold enrichment at both the 6′′′-methyl and the 3′-methoxy carbons was obtained upon feeding with [methyl-^13^C]l-Met, both of which are consistent with *S*-adenosyl-l-methionine (AdoMet) as the biosynthetic precursor of the methyl groups [27].

### 3.2. Identification of the Gene Cluster 

The biosynthetic gene clusters for A-500359s and A-503083s were reported in 2009 and 2010, respectively [41,42]. The gene cluster for the A-500359s was first identified using reverse transcription polymerase chain reaction (RT-PCR) with degenerate primers designed to amplify DNA fragments encoding putative NDP-glucose-4,6-dehydratases (NGDH) [43], despite not having a clear role for such an enzyme activity in A-500359 biosynthesis. Several high, low, and non-producing strains were initially generated by chemical mutagenesis, thus providing a correlation of the abundance of the PCR product versus production of A-500359s. A fragment of a putative NDGH that was over-expressed in the high-producing strains and absent in non-producing strains was subsequently cloned to create a digoxigenin (DIG)-labeled fragment, which was then used to probe a genomic library. Two contiguous cosmids were identified and sequenced to reveal a 65-kb DNA region encoding 38 putative open reading frames (*orfs*) (NCBI Accession No. AB476988). RT-PCR analysis was again utilized to analyze the expression of these 38 *orfs* within the different producing and non-producing strains, revealing that 26 *orfs* were likely involved in A-500359 biosynthesis, regulation, and resistance. The development of a genetic system within the **1** producing strain was unsuccessful. Therefore, to provide additional evidence that the correct genomic locus was identified, one of these *orfs—orf21* encoding a putative aminoglycoside phosphotransferase—was heterologously expressed in **1**-sensitive strains to reveal the gene product confers selective resistance to **1**.

**Figure 4:**
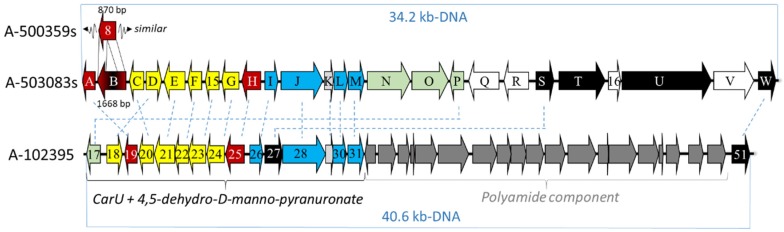
Depiction of the genetic organization of the biosynthetic gene clusters for the capuramycin-type nucleoside.

To provide convincing evidence that the gene cluster for the capuramycin-type nucleoside antibiotics was identified, the gene cluster for the A-503083s was targeted for cloning and characterization [42]. As previously noted, A-503083s—unlike A-500359s—contain a 2′-*O*-carbamoyl group, and it was therefore expected that a functional carbamoyltransferase was encoded within the gene cluster. Interestingly, the gene cluster for A-500359s encodes an *orf* (*orf8*) with sequence similarity to known carbamoyltransferases, but the *orf8* gene product appears to be missing ~50% of the internal sequence when compared to typical carbamoyltransferases. The *orf8* gene remnant—along with another expression-correlated *orf* encoding a putative nonribosomal peptide synthetase—were utilized as probes to isolate four contiguous cosmids from the genomic DNA of the producing strain of A-503083s. Sequencing of the four cosmids revealed ~81 kb-DNA encompassing 55 *orfs* (NCBI Accession No. AB538860), of which 23 gene products annotated as CapA-W had high sequence identity (72% to 91%) with those from the gene cluster of A-500359s (Figure 4 and Table 1). A putative carbamoyltransferase (CapB) was discovered and, as expected, appeared to be full length and functional based on bioinformatics analysis.

Unlike with the producing strain for A-500359s, an attempt to develop a genetic system in the producer of A-503083s was initially successful. The *capU* gene, encoding a tridomain nonribosomal peptide synthetase that was predicted to be involved in l-ACL formation, was inactivated [44]. The corresponding *ΔcapU* strain was unable to produce the l-ACL-containing A-503083 A (**6**) and A-503083 B (**7**) but instead produced relatively high levels of the deaminocaprolactam analog A-503083 F (**11**). Feeding the mutant strain with l-ACL restored the production of **6** and **7**, revealing that *capU* was essential for the biosynthesis of the l-ACL component. Importantly, the results provided the first direct genetic evidence that the genomic island was indeed essential for the biosynthesis of A-503083s. Unfortunately, additional attempts to make other mutations within the producing strain of the A-503083s have failed for unknown reasons.

The **13** gene cluster was identified in 2015 from a cosmid DNA library using a DIG-labeled fragment of the l-Thr:uridine-5′-aldehyde transaldolase gene, which had already been characterized from the gene cluster of the A-500359s and A-503083s and shown to be necessary for the biosynthesis of the CarU moiety [45,46]. Four cosmids were sequenced, revealing 76 *orfs* within ~85-kb genomic DNA (NCBI Accession No. KP995196). Of the identified *orfs*, 40 were proposed to be likely responsible for the biosynthesis of **13**; however, only 16 *orfs* were found to be shared among all three groups of capuramycin-type antibiotics (Table 1). Therefore, it is proposed that 15 *orfs* are involved in the biosynthesis of A-500359E (**8**), the likely last shared intermediate of all three biosynthetic pathways, and one *orf* (*cpr51*) was responsible for the attachment of the polyamide via aminolysis. Similar to the producing strain for A-503083s, a genetic system has been developed within the **13** producing strain. Both *Δcpr25* and *Δcpr51* mutant strains have been independently prepared, and both mutations abolished the production of **13**. However, attempts to isolate biosynthetic intermediates or genetically complement these mutations have so far been unsuccessful.

### 3.3. Functional Assignment of Gene Products 

**Figure 5:**
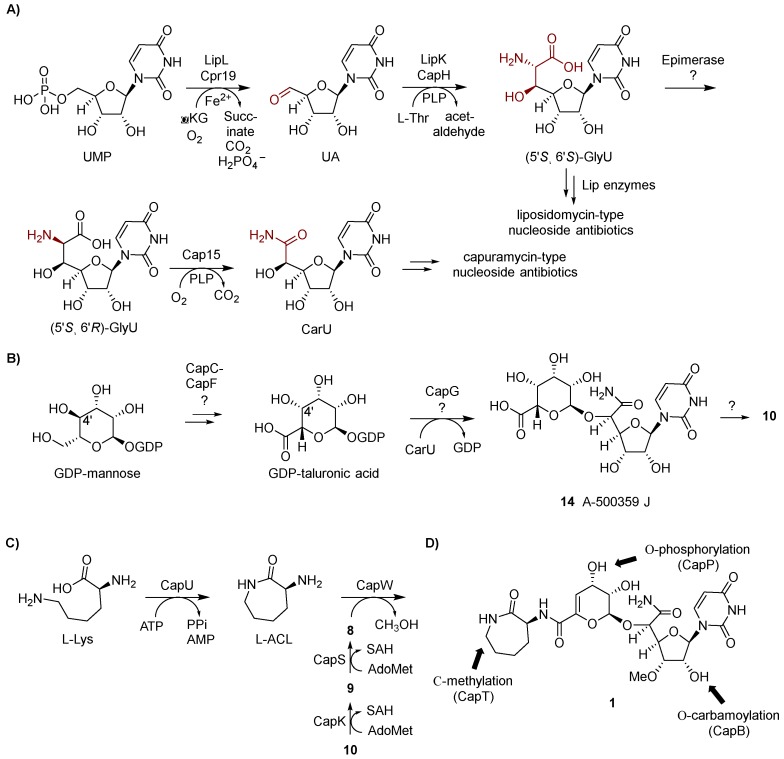
Biosynthetic pathway for the capuramycin-type nucleoside antibiotics. (**A**) Biosynthetic pathway leading to uridine-5′-carboxamide (CarU). αKG, α-ketoglutarate; UA, uridine-5′-aldehyde; PLP, pyridoxal 5′-phosphate; GlyU, 5′-*C*-glycyluridine. (**B**) Proposed biosynthetic pathway leading to the 4,5-dehydro-d-manno-pyranuronate component. (**C**) Biosynthetic pathway leading to the formation and attachment of the l-aminocaprolactam (l-ACL). (**D**) Group transfer reactions that occur after the assembly of the signature capuramycin scaffold.

Insight into the biosynthesis of CarU was obtained from the independent efforts to characterize the biosynthetic mechanism of the GlyU component of the liposidomycin-type nucleoside antibiotics. Utilizing recombinant enzymes from the biosynthetic pathway for A-90289s, GlyU was shown to be derived from UMP via two sequential enzyme-catalyzed reactions (Figure 5A). The first reaction is catalyzed by LipL, a non-heme Fe(II)- and α-ketoglutarate (αKG)-dependent dioxygenase [47]. LipL stereoselectively hydroxylates C-5′ of UMP, resulting in the elimination of phosphate with concomitant formation of the first pathway intermediate, uridine-5′-aldehyde (UA) [48]. Subsequently, LipK, predicted to be a pyridoxal-5′-phosphate (PLP)-dependent serine hydroxymethyltransferase, functions as a transaldolase by catalyzing the conversion of l-Thr and UA to GlyU and acetaldehyde [45]. The LipK-catalyzed reaction was shown to be stereoselective both in the selection of the l-Thr substrate and the formation of (5′*S*,6′*S*)-GlyU. Comparative bioinformatic analysis revealed homologs to both LipL and LipK are encoded within the gene cluster for each group of capuramycin-type antibiotics, suggesting that CarU is derived from UMP by way of (5′*S*,6′*S*)-GlyU. If this is indeed the case, the direct precursor of the carboxamide of CarU would be expected to be l-Thr instead of phosphoenolpyruvate as previously proposed. Consequently, feeding experiments to probe the origin of C-6′ were revisited. As predicted from bioinformatics, l-[^13^C_4_,^15^N]Thr, when fed to the producing strain of the A-503083s, resulted in a 15% enrichment at C-6′ of **7** (Figure 2) [46]. This level of enrichment is comparable to the levels for the enrichment of l-ACL with l-Lys, suggesting that l-Thr is a direct precursor. Furthermore C-N *J* coupling suggested that the C_α_-N bond of l-Thr remained intact during CarU biosynthesis.

With this information in hand, the biosynthesis of CarU was examined using recombinant enzymes. Cpr19, the LipK homolog encoded within the **13** gene cluster, was demonstrated to use Fe(II), O_2_, and αKG to convert UMP to UA (Figure 5A) [46]. CapH and Cpr25, the LipK homologs encoded within the A-503083s and **13** gene clusters, respectively, catalyzed the identical (and expected) chemistry: the conversion of UA to (5′*S*,6′*S*)-GlyU, thus supporting the results from the feeding experiments. The formation of (5′*S*,6′*S*)-GlyU as a pathway intermediate suggested downstream chemistry involving C-6′-decarboxylation—shortening the heptose to a hexose, the introduction of an O atom at C-6′, and oxidation. Given the precedence for PLP-dependent enzymes in catalyzing decarboxylation of α-amino acids, the three gene clusters were analyzed for potential shared candidates with a predicted PLP dependency. This search revealed one unassigned gene product (Cap15 for the biosynthesis of A-503083s) with similarity to bacterial PLP-dependent l-seryl-tRNA (Sec) selemium transferase, which catalyzes the β-replacement of the hydroxyl group of tRNA-loaded l-Ser with Se using selenophosphate as the Se source. However, no reaction was observed when Cap15 was incubated with (5′*S*,6′*S*)-GlyU. Rather, serendipitously, Cap15 was discovered to function as a PLP-dependent monooxygenase-decarboxylase that directly converts (5′*S*,6′*R*)-GlyU directly to CarU in a single reaction (Figure 5A) [49]. The finding that Cap15 is stereoselective for the 6′*R*-isomer of GlyU suggests an epimerase is needed to convert the CapH product to the Cap15 substrate. One potential candidate for this reaction is CapD, an unassigned Fe(II) and αKG-dioxgenase with low sequence similarity to Cpr19. Other members of this large dioxygenase superfamily—for example, CarC—are known to catalyze epimerization at carbon centers bonded to an amine, thereby providing precedence for this putative functional assignment [50,51,52]. Further biochemical characterization of Cap15 revealed other intriguing properties including that (i) the enzyme activates molecular oxygen using a substrate-PLP aldimine as the initial reducing agent, an unusual example of the use of the PLP cofactor as a redox cofactor and (ii) the activity was dependent upon phosphate despite no obvious role in the chemistry [49]. 

The enzymes required for the biosynthesis of the α-d-*manno*-pyranuronate component of the capuramycin-type nucleoside antibiotics remain to be functionally assigned. If the moiety is derived from d-mannose as the feeding experiments would suggest, the most direct path would involve a four-electron oxidation of C-6, a 4,5-dehydration, and glycosyltransfer to CarU. Analysis of the biosynthetic gene clusters revealed several gene products with similarity to enzymes involved bacterial sugar biosynthetic pathways, including a putative glycosyltransferase (CapG for A-503083) that could catalyze the last step. However, it is not obvious how the other gene products, namely CapC, CapE, and CapF for A-503083s, would contribute to the expected chemistry, which we propose begins from the precursor GDP-mannose (Figure 5B). Nonetheless, the isolation of a taluronic acid-containing congener of A-500359 H (**10**) called A-500359 J (**14**) suggests 4,5-dehydration occurs after CapG-catalyzed transfer to the acceptor, CarU [20]. Additionally, this pathway would necessitate C-4′ epimerization to generate GDP-taluronic acid, although it is not clear whether this occurs before or after glycosyltransfer. As previously noted, the NDGH-like protein is not expected to play a role in α-d-*manno*-pyranuronate biosynthesis since (i) a 4,6-dehydration catalyzed by the canonical NDGH is apparently not needed and (ii) an NDGH homolog is not encoded within the **13** gene cluster.

The formation and attachment of the l-ACL moiety of the A-500359s and A-503083s has been defined using enzymes from the biosynthetic pathway for A503083s, revealing two different mechanisms for amide bond formation. The initial step in the biosynthesis involves a traditional ATP-dependent reaction catalyzed by CapU: adenylation and transfer of l-Lys to the sulfhydryl group of the carrier protein domain located at the C-terminus of CapU followed by intramolecular aminolysis to release the lactam, the latter step of which appears to be nonenzymatic (Figure 5C) [44]. The subsequent step, the attachment of the l-ACL, involves an unusual ATP-independent transacylation mechanism [42]. CapS first catalyzes an AdoMet-dependent carboxyl methylation of **9** to generate the methyl ester A-500359 E (**8**). This reaction effectively “activates” the carboxylate such that CapW—a protein with sequence similarity to AmpC β-lactamases that employ an active site Ser residue to generate an acyl enzyme intermediate during the reaction coordinate—catalyzes transacylation to produce methanol and the l-ACL-containing **1**. Despite a dramatic difference in the structure, the aryl-containing polyamide of **13** is expected to be attached in a similar manner due to the uncovering of homologs of the carboxylmethyltransferase (Cpr27) and transacylase (Cpr51) [46]. However, enzymes catalyzing these putative reactions or any reactions involved in the biosynthesis of the polyamide of **13** have been characterized to date. Finally, we propose that O-methylation of the C-3′-hydroxyl is catalyzed by CapK and occurs prior to the attachment of the l-ACL since compounds **8** and **9** are isolated as the major congeners. However, the l-ACL-containing congener A-500359 G (**5**) is produced in small amounts [24], suggesting either the amide coupling enzymes have relaxed specificity toward this modification or the methylation can occur post l-ACL attachment.

Three enzyme-catalyzed group transfer reactions occur following the synthesis of **1**, two of which have been characterized in vitro (Figure 5D). CapB, the predicted full-length carbamoyltransferase that was used in part to identify the biosynthetic gene cluster for the A-503083s, catalyzes the carbamoyl phosphate-dependent addition of a carbamoyl group to the C-2′ hydroxyl to convert **1** to **7** (assuming this “tailoring” step occurs first) [42]. CapT, a putative radical SAM C-methyltransferase, likely catalyzes the stereoselective methylation of the l-ACL, thereby converting **7** to **6** (assuming this reaction follows CapB). Finally, CapP was functionally assigned as an ATP-dependent **7** 3′′-*O*-phosphotransferase that was shown to likely confer self-resistance to the producing strain [53]. Cpr17, the CapP homolog in the **13** biosynthetic pathway, was similarly demonstrated to have an identical phosphotransferase function [54]. Nonetheless, phosphorylated capuramycin analogs have not been isolated from the producing strains. 

## 4. Chemical and Enzymatic Synthesis of Capuramycin and Analogs

Since the structural elucidation of capuramycin in 1988 [33], a few total syntheses have been independently reported. Moreover, a large library of analogs prepared via semisynthesis, total synthesis, and biocatalysis have been screened for antibacterial activity. These efforts, which have resulted in the advancement of a few lead compounds for further development, are described below.

### 4.1. Total Synthesis 

**Figure 6:**
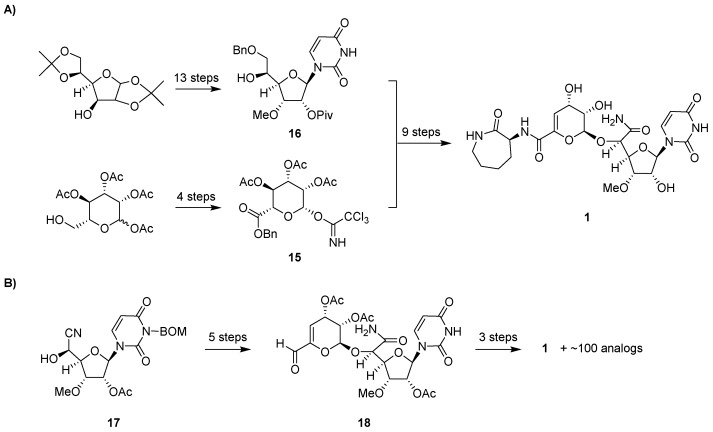
Total synthesis of **1**. (**A**) Strategy for the first reported total synthesis. (**B**) Strategy for the more recently reported synthesis using significantly fewer steps, which has enabled the synthesis of capuramycin analogs. BOM, benzyloxymethyl acetal.

The total synthesis of **1** was first reported in 1994 (Figure 6) [55]. This synthesis utilized a mostly linear, 22-step approach involving convergence of an α-d-*manno*-pyranuronate sugar donor **15** and protected l-talo-uridine as the sugar acceptor **16**. The synthesis of **16** started from diacetone glucose, going through a 13-step sequence with a 27% overall yield. Acetyl-protected mannopyranose was used to prepare **15** in four steps. The synthesized product confirmed the proposed structure including the absolute configuration of **1** that was initially deduced upon structural characterization of the natural product. An improved total synthesis of **1** was reported in 2009 using 15 linear steps [56]. Starting from the cyanohydrin intermediate **17**, **1** was synthesized in eight steps with overall 30% yield utilizing the aldehyde derivative **18** as an intermediate. The synthesis was further improved by adopting a new protecting group—a monomethoxydiphenylmethoxymethyl (MDPM) group [57]—that is more stable under diverse conditions and can be adequately removed in 30% trifluoroacetic acid [58]. 

### 4.2. Preparation of 1 Analogs

The synthesis of analogs of **1** (Table 2) was initially pursued shortly after the discovery of **1** to **12** using a semisynthetic strategy to substitute the l-ACL component by starting with congener **8** [59]. With strong nucleophilic amines, a single-step aminolysis reaction afforded the products in decent yields. Alternatively, a multistep protection-deprotection sequence that included saponification and condensation was utilized. Sixty-eight capuramycin analogs, represented by **19** to **26**, were prepared. A few of these analogs displayed improved TL1 inhibition and antibacterial activity against various *Mycobacterium* species including *M. smegmatis*, *M. avium*, *M. intracellulare*, and *M. kansasii* [31]. Of the analogs tested, **24** (compound 65 in the original paper), which has a 3,4-difluoroaniline in place of the l-ACL, showed improved MIC values (0.5 to 2 µg/mL compared to 8 to 12.5 µg/mL for **1**).

Additional studies focused on the modification of the 2′-hydroxyl of **1** and **2** by introducing different acyl, alkoxycarbonyl, or alkylether groups in three to five steps (Table 2) [60]. Fifty-one analogs were synthesized and tested for antimycobacterial activity with a few showing improved activity compared to the parent compounds (Table 2). Among them, **28** (compound 20 in the original paper), which has a decanoyl chain, showed improved MIC against the four species of *Mycobacterium*. Interestingly, **28** was 55-fold less potency against TL1 when compared to the parent compound, **2**. The improved MIC of **28** was hypothesized to be due to the increased lipophilicity that results in the effective penetration of the *Mtb* cell wall, thus compensating for the decrease in IC_50_ (assuming the acyl group remains intact, which remains to be determined). Indeed, the penetration of *Mtb* cell walls has been a major challenge in the field of anti-TB drug discovery, and comparable acylation strategies for other drug classes of potential anti-TB drugs have been shown to have a similar effect [61,62].

In total, over 7000 capuramycin analogs were synthesized using different combinations of the aforementioned semisynthetic approaches and screened for antimycobacterial activity [63,64], ultimately yielding **24** (named RS-124922 by Sankyo Co.; renamed to SQ922 by Sequella Inc. after licensing the library), and **28** (RS-118641; SQ641) as leads [65,66]. In comparison to parent **2** (RS-112997; SQ997), compound **28** displayed a significantly improved antimycobacterial activity with MICs as follows: 1 μg/mL against non-MDR *Mtb* (n = 22 clinical isolates; 16 μg/mL for **2**), 0.5 μg/mL against MDR-*Mtb* (n = 11; 16 μg/mL for **2**), 4 μg/mL against *M. avium* (n = 33; 8 μg/mL for **2**), and 0.06 μg/mL against *M. intracellulare* (n = 17; 2 μg/mL for **2**) [65]. By further examining the activity against *Mtb* H37Rv, compounds **24** and **28** were shown to kill faster than other clinically used anti-TB drugs including isoniazid and rifampicin [65]. Both analogs appear to induce bacteriolysis of *Mtb,* and **28** had a clear post-antibiotic effect that lasted as long as 55 h. Compound **28** also showed synergistic activity with other anti-TB drugs, including streptomycin and ethambutol [65]. Finally, when formulated with a vitamin E derivative or as micelles and tested in vivo, **28** displayed ~2 log reduction in burden using a murine lung model of *Mtb* H37Rv infection [66]. No noticeable toxicity was observed.

**Figure 7:**
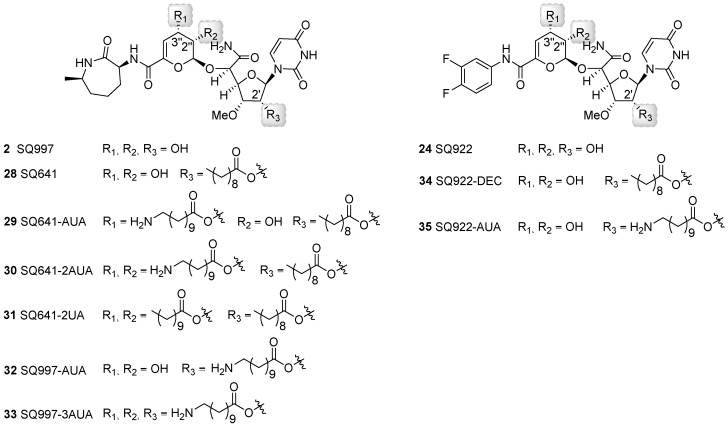
Structures of capuramycin analogs generated through semisynthesis and modified with decanoic acid (DEC), undecanoic acid (UA), and/or aminoundecanoic acid (AUA).

The impact of different long chain ester modifications at the 2′, 2′′, and/or 3′′ positions of **2**, **24**, and **28** has been explored as a strategy to improve activity against mycobacteria and extend the antibiotic spectrum (Figure 7) [67,68]. Promising results were obtained when conjugated with decanoic acid (DEC) or amino undecanoic acid (AUA) exemplified by compounds **29** to **35**. With respect to the antibiotic spectrum, compounds **28**-3′′-AUA (**29**), **2**-2′,2′′,3′′-triAUA (**33**), and **24**-2′-DEC (**34**) showed a gain in the spectrum as desired, generating compounds active against *Escherichia coli*, *Pseudomonas aeruginosa*, methicillin-resistant *Staphylococcus aureus*, and/or vancomycin-resistant *Enterococcus* [67]. The activity against *Staphylococcus aureus* was further improved by co-administration with phenylalanine arginine β-naphthyl amide, an efflux pump inhibitor. The addition of ethylenediaminetetraacetic acid elicited similar improvements to activity against Gram-negative bacteria. The conjugation of AUA in general enhanced the intracellular killing against *Mtb* H37RV, although the overall effect was minimal. Nonetheless, it was clear from these studies that AUA moieties were superior to DEC; for example, conjugation of two AUA moieties on **28** (**30**) led to a slight decrease of antimycobacterial activity whereas conjugation of two undecanoic acid (UA) moieties to generate **31** caused complete loss of antibacterial activity [67]. This result suggested that the additional amino groups on the AUA moieties improve the permeability of capuramycin and/or affect drug efflux. The metabolic fate of the acyl chains—which might be prone to hydrolysis by the numerous *Mtb* esterases that have been recently characterized [62,69]—awaits further testing. 

More recently, **28** was demonstrated to be effective in *Clostridium difficile* infection (CDI) treatment in a murine model [70]. Although treatment with **28** at >1 mg/kg/day for 5 days was shown to have similar efficacy to vancomycin—a first-line therapy for CDI—at 20 mg/kg/day during the initials stages of infection, the post-antibiotic effects such as mice survival rates and reduced weight loss were significantly improved with **28**.

**Figure 8:**
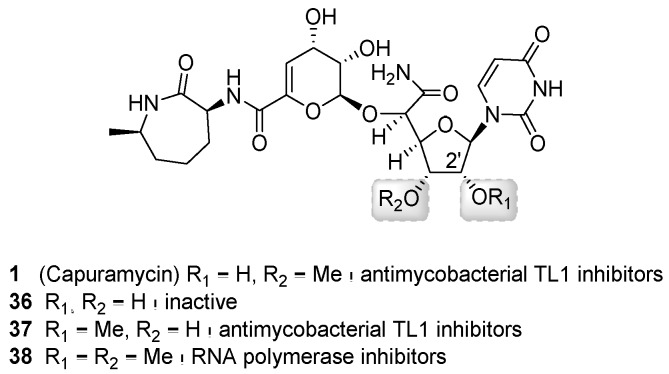
Structures of capuramycin analogs generated through total synthesis and a summary of structure–activity relationship.

In addition to a semisynthetic approach, total synthesis has also been successfully used to generate novel capuramycin analogs for structure-activity relationship studies (Figure 6B) [58,71,72]. Of the ~100 analogs prepared, perhaps the most promising result has been achieved by examining the effects of differential O-methylation of the ribose of the CarU component (Figure 8). The absence of the 3′-*O*-methyl group of **1** yielded an inactivate analog (**36**). Swapping the position of the methyl group to generate the 3′-desmethyl, 2′-*O*-methyl-**1** (**37**) yielded an analog with similar TL1 inhibitory activity and antimycobacterial activity compared to **1**. However, neither **1** nor **37** was effective against dormant *Mtb*. Contrastingly, the 2′-*O*,3′-*O*-dimethyl-**1** (**38**, named UT01320) not only killed replicating but also nonreplicating *Mtb*. Compound **38** unexpectedly did not inhibit TL1 but instead inhibited RNA polymerase from *M. smegmatis* and *S. aureus* with the IC_50_ values of 0.1 to 0.15 μM [72]. Furthermore, **38** and **24** were strongly synergistic, suggesting that inhibiting these two distinct targets is a promising approach warranting additional studies.

**Figure 9:**
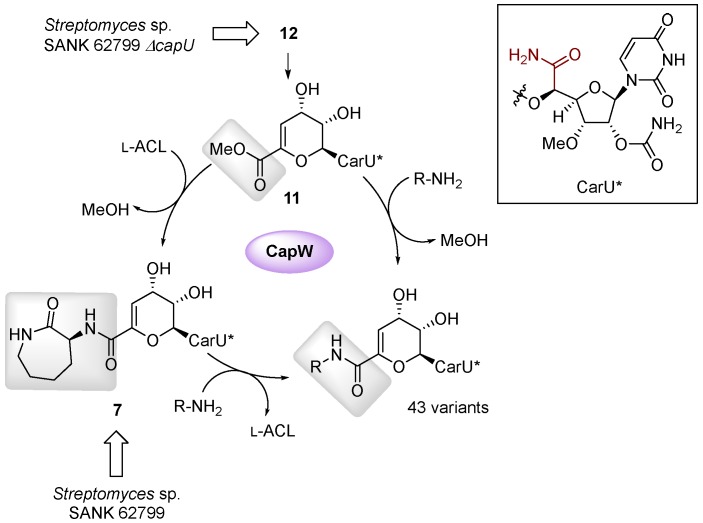
Biocatalytic approach used to synthesize capuramycin analogs.

Finally, a biocatalytic approach has been reported as a complimentary strategy to semi- and total-synthetic efforts [54]. This approach took advantage of the discovery of the enzymatic activity of CapW, a transacylase that utilizes the methyl ester of **11** as an acyl donor and l-ACL as the acyl acceptor to generate a new amide bond (Figure 9). Compound **12** was isolated as the major congener from the *ΔcapU* mutant strain and converted to **11** via chemical or enzymatic synthesis. Subsequently, incubation of CapW with **11** and different amine acceptors gave the corresponding analogs in modest yields. Amine acceptors with carboxylate functionalities (for example, α-amino acids) were not incorporated by CapW; however, this limitation could be overcome by using methyl esters or Boc-protected α-amino acids. Further interrogation into the biochemical properties of CapW revealed the enzyme not only performed an amide–ester exchange reaction but could also catalyze a direct transamidation reaction with **7**—isolated with decent yields from the wild-type strain—as the acyl donor, thereby substituting the l-ACL in a single-step reaction. Using both acyl exchange strategies, a total of 43 analogs were synthesized using nonnative amines as acyl acceptors. Biological activity assays indicated that a few of the analogs had significantly improved antibacterial activity against *M. smegmatis* and *Mtb* H37Rv. 

## 5. Prospects

The re-discovery of the capuramycin scaffold, the parent of which was first isolated in 1986 based on antibacterial activity, has sparked significant efforts aimed at preparing analogs for antibiotic discovery and development. To date the most fruitful results have been achieved using semisynthetic modification of the parent natural product with an emphasis on l-ACL substitution starting from **8** or acylation of the reactive hydroxyl groups of **2**. The semisynthetic strategy has led to the discovery of a few analogs such as **24** and **28** that have superior activity against *Mtb* in comparison to the parent. Furthermore, some of the analogs have an extended spectrum of activity, suggesting the development of capuramycin-type nucleoside antibiotics is not limited to mycobacteria. Formulation and delivery studies [73,74,75,76] have further improved the solubility and pharmacodynamics of some of these lead compounds, thereby pushing this family further down the translational pipeline. 

Total synthesis and enzymatic synthesis have more recently emerged as two alternative strategies to generate capuramycin analogs. Importantly, both strategies afford an opportunity to probe the structure–activity relationship of core components of the capurmaycin scaffold in greater detail. The former approach has enabled the synthesis of new l-ACL-substituted analogs and the dimethylated analog **38**. The latter approach has enabled a chemoenzymatic strategy to likewise synthesize new l-ACL-substituted analogs. Unfortunately, most of the enzyme-catalyzed steps involved in capuramycin biosynthesis remain undefined, particularly with respect to the formation and attachment of the α-d-*manno*-pyranuronate. As our understanding of the biosynthetic pathway improves, it is expected that capuramycin scaffolds altered within the α-d-*manno*-pyranuronate or elsewhere can be readily prepared through enzymatic synthesis, thereby generating a new array of analogs that will be valuable for continued anti-TB drug discovery efforts.

## Figures and Tables

**Table 1:** Shared *orfs* within the cloned biosynthetic gene clusters.

Proposed Function	A-500359	A-503083	A-102395
Fe(II)- and αKG-dependent dioxygenase	*orf7*	*capD*	*cpr18*
Capuramycin-2′-*O*-carbamoyltransferase	*orf8*	*capB*	*NA*
Putative 3-ketoreductase	*orf9*	*capC*	*cpr20*
Fe(II)-dependent, αKG:UMP dioxygenase	*orf10*	*capA*	*cpr19*
Putative 2,3-dehydratase	*orf11*	*capE*	*cpr21*
Putative 4-epimerase	*orf12*	*capF*	*cpr22*
PLP-dependent monooxygenase-decarboxylase	*orf12’*	*cap15*	*cpr23*
Putative glycosyl transferase	*orf13*	*capG*	*cpr24*
l-Thr:uridine-5′-aldehyde transaldolase	*orf14*	*capH*	*cpr25*
Putative pyrophosphatase	*orf15*	*capI*	*cpr26*
Putative CO dehydrogenase	*orf16*	*capJ*	*cpr28*
Putative O-methyltransferase	*orf16’*	*capK*	*cpr29*
Putative CO dehydrogenase	*orf17*	*capL*	*cpr30*
Putative CO dehydrogenase	*orf18*	*capM*	*cpr31*
Putative ABC transporter	*orf19*	*capN*	*NA*
Putative ABC transporter	*orf20*	*capO*	*NA*
Capuramycin 3′′-phosphotransferase	*orf21*	*capP*	*cpr17*
UDP-glucose-4,6-dehydratase	*orf22*	*capQ*	*NA*
Glucose-1-phosphate thymidylyltransferase	*orf23*	*capR*	*NA*
Putative carboxyl methyltransferase	*orf24*	*capS*	*cpr27*
Putative l-ACL C-methyltransferase	*orf25*	*capT*	*NA*
Nonribosomal peptide synthetase	*orf26*	*capU*	*NA*
Nonribosomal peptide synthetase	*orf27*	*capV*	*NA*
Transacylase	*orf28*	*capW*	*cpr51*

**Table 2:** Antimycobacterial activities of several capuramycin analogs prepared through semisynthesis.

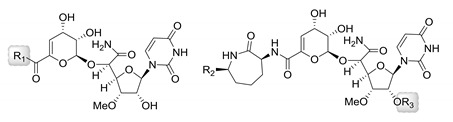								
				TL1 IC_50_ (ng/mL)	MIC (µg/mL) ^1^			
Compound	R_1_	R_2_	R_3_		Strain A	Strain B	Strain C	Strain D
**20**	PhNH-	-	-	6.5	6.25	16	4	8
**21**	PhN(Me)-	-	-	7.6	12.5	4	1	8
**22**	3-F-PhNH-	-	-	10	6.25	2	2	8
**23**	4-F-PhNH-	-	-	37	6.25	4	2	2
**24**	3,4-F_2_-PhNH-	-	-	9	6.25	2	0.5	1
**25**	4-Cl-PhNH-	-	-	18	6.25	4	2	16
**26**	4-Br-PhNH-	-	-	20	6.25	8	0.5	8
**27**	-	H	H(CH_2_)_11_CO-	n.d.	3.13	<0.063	0.125	0.125
**28**	-	Me	H(CH_2_)_9_CO-	550	6.25	<0.063	<0.063	<0.063
**1**				10	12.5	8	8	8
**2**				10	6.25	8	4	16
RIF ^2^					n.d.	0.125	0.125	0.25
IZD ^3^					n.d.	1	8	2

^1^ Strain A: *M. smegmatis* SANK75075; strain B: *M. avium* NIHJ1605; strain C: *M. intracellulare* ATCC19543; strain D: *M. kansasii* ATCC12478; ^2^ Rifampicin; ^3^ Isoniazid
